# The maturational process of the auditory system in the first year of life characterized by brainstem auditory evoked potentials

**DOI:** 10.1590/S1678-77572009000700010

**Published:** 2009

**Authors:** Raquel Beltrão AMORIM, Raquel Sampaio AGOSTINHO-PESSE, Kátia de Freitas ALVARENGA

**Affiliations:** 1 BS, Audiologist of the Speech-Language Pathology and Audiology Clinic and Student of the Master's Program in Speech-Language Pathology and Audiology, Bauru School of Dentistry, University of São Paulo, Bauru, SP, Brazil.; 2 BS, MSc, PhD, Associate Professor, Department of Speech-Language Pathology and Audiology, Bauru School of Dentistry, University of São Paulo, Bauru, SP, Brazil.

**Keywords:** Auditory brainstem evoked responses, Infant, Neuronal plasticity

## Abstract

The study of brainstem auditory evoked potentials (BAEP) allows obtaining the electrophysiological activity generated in the cochlear nerve to the inferior colliculus. In the first months of life, a period of greater neuronal plasticity, important changes are observed in the absolute latency and inter-peak intervals of BAEP, which occur up to the completion of the maturational process, around 18 months of life in full-term newborns, when the response is similar to that of adults. Objective: The goal of this study was to establish normal values of absolute latencies for waves I, III and V and inter-peak intervals I-III, III-V and I-V of the BAEP performed in full-term infants attending the Infant Hearing Health Program of the Speech-Language Pathology and Audiology Course at Bauru School of Dentistry, Brazil, with no risk history for hearing impairment. Material and Methods: The stimulation parameters were: rarefaction click stimulus presented by the 3^A^ insertion phone, intensity of 80 dBnHL and a rate of 21.1 c/s, band-pass filter of 30 and 3,000 Hz and average of 2,000 stimuli. A sample of 86 infants was first divided according to their gestational age in preterm (n = 12) and full-term (n=74), and then according to their chronological age in three periods: P1: 0 to 29 days (n=46), P2: 30 days to 5 months 29 days (n=28) and P3: above 6 months (n= 12). Results: The absolute latency of wave I was similar to that of adults, generally in the 1st month of life, demonstrating a complete process maturity of the auditory nerve. For waves III and V, there was a gradual decrease of absolute latencies with age, characterizing the maturation of axons and synaptic mechanisms in the brainstem level. Conclusion: Age proved to be a determining factor in the absolute latency of the BAEP components, especially those generated in the brainstem, in the first year of life.

## INTRODUCTION

The research of brainstem auditory evoked potentials (BAEPs) allows obtaining the electrical activity generated in the cochlear nerve up to the brainstem through stimulation, with the recording of five waves. Waves I and II are generated in the cochlear nerve^6^, wave III, in the neurons which emerge from the complex of cochlear nuclei^9^^,^^10^^,^^19^, waves IV and V, in the upper lateral lemniscus, the latter followed by a negative contingent termed slow negative 10 (SN10) deriving from the depolarization of the inferior colliculus^7^^,^^18^.

The auditory system presents maturational and developmental patterns that are reflected in the possibility of recording the amplitude, measured in micro-volts (Ωv), and the latency, measured in milliseconds (ms), of the auditory evoked potentials (AEP). Electrophysiological studies for the auditory system have demonstrated that the maturation of the structures occurs from the periphery to the core, without following a hierarchical pattern^3^^,^^14^. In the first months of life, a period of greater neuronal plasticity, important changes are observed in the absolute latency and inter-peak intervals of BAEP, which occur up to the completion of the maturational process, around 18 months of life, in full-term newborns, when the response is similar to that of adults.

In the clinical practice, BAEP analysis is performed by the latencies of waves I, III and V, and values of inter-peak I-III intervals, which reflects the functional state of the hearing nerve and low region of the brainstem. While III-V reflects the higher and central region, I-V encompasses the structure of both intervals^8^.

It is thus possible, through the BAEP research, to evaluate the maturation of the auditory nerve and brainstem, and verify the occurrence of an abnormal development process in preterm newborns or with risk indicators^5^^,^^8^^,^^16^. Hence, the absolute latency and the inter-peak intervals must be precisely determined for each period of development and according to the evaluation protocol utilized, since the BAEP are exogenous potentials, totally dependent on the characteristics of the stimulus utilized to evoke the response.

This study aimed at characterizing the changes in absolute latencies and inter-peaks of the BAEP generated by click stimulus, in the first year of life of normal infants.

## MATERIAL AND METHODS

After approval by the Ethics Committee of Bauru Dental School, University of São Paulo (Protocol #114/2005), this transversal cohort study analyzed absolute latencies for waves I, III and V and inter-peak intervals I-III, III-V and I-V of the BAEP performed in infants with no risk history for hearing impairment attending the Infant Hearing Health Program of the Speech-Language Pathology and Audiology Course at Bauru School of Dentistry. The normal peripheral hearing was determined by means of a battery of tests, carried out according to the period, including otoacoustic emissions, immittance measures, visual reinforcement audiometry and evaluation of the hearing behavior. A sample of 86 infants was first divided according to their gestational age in preterm (n=12) and full-term (n=74), and then according to their chronological age in three periods: P1: 0 to 29 days (n=46), P2: 30 days to 5 months 29 days (n=28) and P3: above 6 months (n= 12). For BAEP analysis, the rarefaction click stimulus was presented by the 3 Ω insertion phone, with intensity of 80 dBnHL and a presentation rate of 21.1 c/s, with a band-pass filter of 30 and 3,000Hz and average of 2,000 stimuli, Navigator Pro Bio-logic System Corp, version 4.2.0. The BAEP were captured through ECG disposable electrodes (MEDITRACE_TM_ 200), with EEG conductive paste (Tern 20_TM_), placed after cleaning the skin with ECG/EEG abrasive gel (NUPREP). The impedance level was kept between 1 and 3 KΩ for the electrodes: the active electrode was positioned in F_z_, the reference electrodes in M_1_ and M_2_, and the ground electrode in Fpz, which allowed the ipsilateral and contralateral recording of the response.

For statistical purposes, a descriptive analysis of the variables was done, and the Student's t-test and two-way analysis were used. A significance level of 5% was set for all analyses.

## RESULTS

The result of the Student's t-test for comparison between the right and left ears of all infants showed no statistically significant difference for either the absolute latencies (wave I: p=0.717; wave III: p=0.883; wave V: p=0.384) or the inter-peak interval values (I-III: p=0.105; III-V: p=0.375; and I-V: p=0.573). Thus, statistical analysis was carried out taking into account the individual and not the ears separately.

Tables 1 and 2 present the descriptive analysis (mean and standard deviation) of the absolute latencies for waves I, III and V and values of inter-peak intervals I-III, III-V and I-V, respectively, according to the gestational age and analysed periods.

**Table 1:** Descriptive analysis of absolute latencies for waves I, III and V for groups of full-term and preterm infants, according to the gestational age and period

	ABSOLUTE LATENCIES					
GA/P	I		III		V	
	Mean	SD	Mean	SD	Mean	SD
Preterm/P1	1.80	0.35	4.47	0.75	6.66	0.55
Preterm/P2	1.60	0.20	4.26	0.18	6.32	0.24
Preterm/P3	1.62	0.20	4.09	0.27	6.23	0.29
Full term/P1	1.67	0.28	4.49	0.47	6.77	0.54
Full term/P2	1.71	0.30	4.32	0.33	6.50	0.33
Full term/P3	1.71	0.20	3.97	0.28	6.23	0.30

GA: gestational age; P: Period; SD: standard deviation

**Table 2:** Descriptive analysis for inter-peak interval values I-III, III-V and I-V, for the groups of full-term and preterm infants, according to the gestational age and period

	INTER-PEAK INTERVAL VALUES					
GA/P	I-III		III-V		I-V	
	Mean	SD	Mean	SD	Mean	SD
Preterm/P1	2.66	0.43	2.19	0.22	4.85	0.28
Preterm/P2	2.66	0.21	2.06	0.19	4.72	0.26
Preterm/P3	2.48	0.20	2.14	0.23	4.61	0.25
Full term/P1	2.80	0.49	2.25	0.50	5.05	0.75
Full term/P2	2.61	0.18	2.18	0.20	4.79	0.26
Full term/P3	2.12	0.64	2.49	1.14	4.61	0.54

GA: gestational age; P: Period; SD: standard deviation

The results of the two-way analysis of variance for comparison of the variables gestational age, period and their interaction for the absolute latencies of waves I, III and V and inter-peak interval values I-III, III-V and I-V, are presented in Table 3. Table 4 shows the results of Tukey's test for the absolute latencies of waves III and V and inter-peak I-III interval value.

**Table 3:** Results of the two-way analysis of variance for comparison of the variables gestational age and period, and their interaction for the absolute latencies and inter-peak intervals

	p Value		
Wave/lnterval	Gestational age	Period	Interaction
I	0.666	0.574	0.299
III	0.925	0.008*	0.720
V	0.400	0.010*	0.762
I-III	0.413	0.007*	0.241
III-V	0.180	0.394	0.664
I-V	0.558	0.254	0.879

* p<0.05: statistically significant.

**Table 4:** Results of Tukey's test for absolute latencies of waves III and V and inter-peak I-III interval value

Wave	P1 (0 to 29 d)	P2 (30 d to 5 m29 d)	P3 (>6 m)
III	4.48^a^	4.29^b^	4.03^c^
V	6.71^a^	6.41^b^	6.23^b^
I-III	2.73^a^	2.63^a^	2.30^b^

P: Period; Same letters in the columns indicate no statistically significant difference at 5%; d= days; m= months

**Figure 1:**
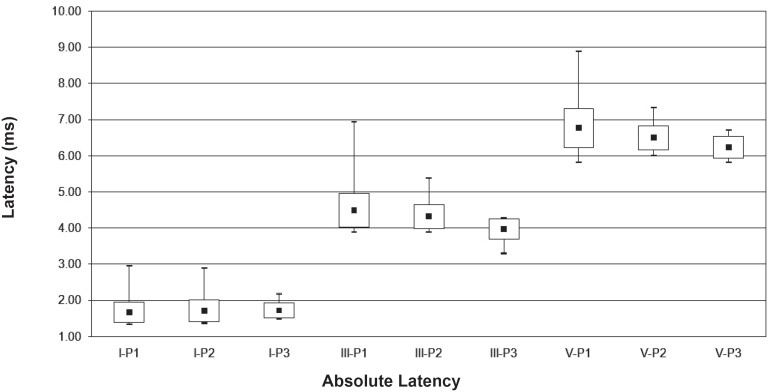
Mean, minimum, maximum and standad deviation values of absolute latencies for waves I, III and V, for the full- term infants according to the period

**Figure 2:**
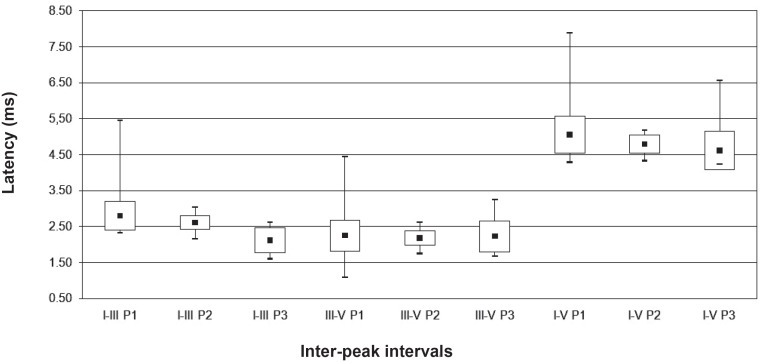
Mean, minimum, maximum and standad deviation values of inter-peak I-III, III-V and I-V interval values, for the full-term infants according to the period

**Figure 3:**
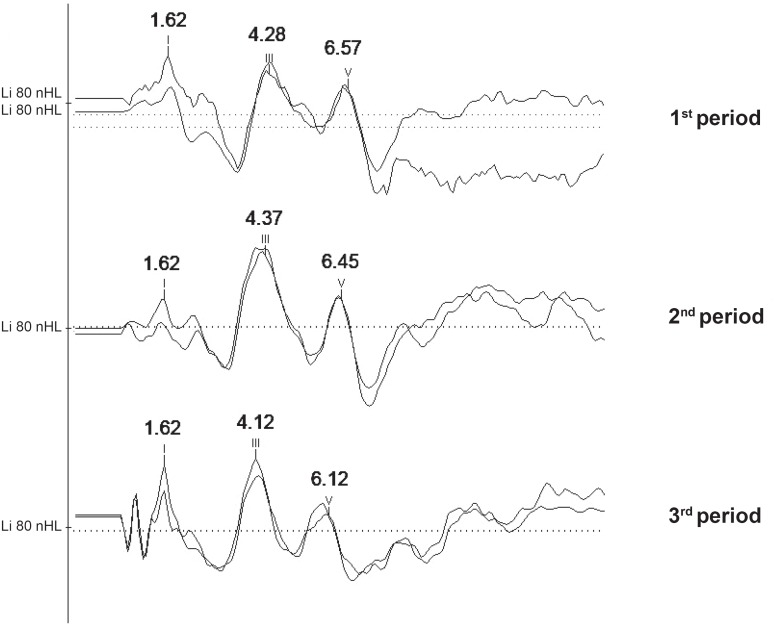
Recording of brainstem auditory evoked potentials (BAEP) with the respective latencies for waves I, III and V, of inter-peak interval values I-III, III-V and l-V, for the full-term infants according to the period

Due to the reduced casuistic of the preterm group, the normality characterization was performed taking into account the full-term group. Figures 1 and 2 present the minimum, maximum, mean and SD values of absolute latencies for waves I, III and V, and inter-peak interval values I-III, III-V and I-V, respectively, obtained in the in full-term infants according to the period. Figure 3 presents the recorded BAEP with the respective absolute latencies for waves I, III and V, and values of inter-peak intervals I-III, III-V and I-V, in the three periods analysed.

## DISCUSSION

In the present study, there was no statistically significant difference between the right and left ears for the absolute latencies and inter-peak values, which indicates that the maturational process occurs in a similar manner in both, with no inter-aural difference, corroborating the data in the literature^1^^,^^17^.

No difference was seen for the absolute latencies of waves I, III and V, and the inter-peak interval values, when comparing full-term and preterm infants (Table 3). This finding must be analyzed with caution due to the small sample size in the preterm group. However, this similar behavior of absolute latencies and inter-peak values in preterm and term children has been described^4^, though it is not consistent with other studies^2^^,^^17^.

There was no significant difference (p=0.666) for the absolute latency of wave I among periods analyzed in this study (Table 3). The absolute latency of wave I was similar to that of adults (1.67±0.28 ms), already in the first period studied, remaining similar in the subsequent periods, demonstrating that the maturational process of the distal portion of the auditory nerve is practically complete in the first month of live^5^^,^^12^ (Table 1). Clinically, this is an important information since the delay in the absolute latency of wave I might aid the clinician in determining the presence of alteration in the peripheral function, involving the middle and/or inner ear.

On the other hand, the absolute latencies of waves III and V and inter-peak values I-III, III-V and I-V tended to diminish as the period increased (Tables 1 and 2), with a significant correlation for wave III (p=0.008) in P2 (p=0.015) and P3 (p=0.000), and between P2 and P3 (p=0.032); for wave V (p=0.009) in P2 (p=0.000) and P3 (0.000), and for interval I-III (p=0.006) in P3 (p=0.000), and between P2 and P3 (p=0.004), characterizing the myelinization of axons and maturation of the synaptic mechanisms at the brainstem level^8^^,^^11^^,^^15^. The absolute latency of wave III showed to be similar to that of adults, in the third period, 4.09±0.27 ms for the preterm group and 3.97±0.28 ms for the full- term group. This result demonstrates that the maturational process in the region of the cochlear nucleus in the lower portion of the brainstem is complete, in the first year of life. However, the lateral lemniscus area, the upper portion of the brainstem, represented by wave V, 6.23±0.29 ms for the preterm group and 6.23±0.30 ms for the full-term group, will keep its development in the second year of life^13^^,^^15^. These findings confirm that the maturation is peripheral-central/ caudal-rostral, occurring in different speeds in the structures of the brainstem and in different phases of development^5^^,^^8^^,^^17^.

This way, the following values of absolute latencies for each period, expressed in ms, respectively, can be adopted for the analysis of BAEP recording of full-term infants: wave I - 1.67±0.28/1.71±0.30/1.72±0.20; wave III - 4,49±0.47/4,32±0.33/3.97±0.28; wave V - 6.77±0.54/6.50±0.33/6,23±0.30, with a proportional decrease of inter-peak values I-III 2.80±0.49/2.61±0.18/2,12±0.64, III-V 2.25±0.50/2,18±0.20/2.49±1.14 and I-V 5.05±0.75/4.79±0.26/4.61±0.54.

The knowledge of this process is determinant for the speech pathologist to undertake an accurate analysis of the brainstem auditory evoked potential accomplished in full-term infants.

## CONCLUSION

Age was proven to be determinant in the absolute latency and inter-peak interval values of the brainstorm auditory evoked potentials (BAEP) components, especially those generated in the brainstem, within the first year of life.
